# Pre- and peri-implantation Zika virus infection impairs fetal development by targeting trophectoderm cells

**DOI:** 10.1038/s41467-019-12063-2

**Published:** 2019-09-13

**Authors:** Lei Tan, Lauretta A. Lacko, Ting Zhou, Delia Tomoiaga, Romulo Hurtado, Tuo Zhang, Ana Sevilla, Aaron Zhong, Christopher E Mason, Scott Noggle, Todd Evans, Heidi Stuhlmann, Robert E. Schwartz, Shuibing Chen

**Affiliations:** 1 Department of Surgery, Weill Cornell Medical College, 1300 York Ave, New York, NY 10065 USA; 2 Department of Cell and Developmental Biology, Weill Cornell Medical College, 1300 York Ave, New York, NY 10065 USA; 3 The SKI Stem Cell Research Facility, The Center for Stem Cell Biology and Developmental Biology Program, Sloan Kettering Institute, 1275 York Avenue, New York, NY 10065 USA; 4 Physiology Biophysics and Systems Biology, Weill Cornell Medical College, 1300 York Ave, New York, NY 10065 USA; 5 Genomics Resources Core Facility, Weill Cornell Medical College, 1300 York Ave, New York, NY 10065 USA; 6 New York Stem Cell Foundation, 619 W 54th St, New York, NY 10019 USA; 7 Department of Physiology, Cellular Biology and Immunology, Faculty of Biology, University of Barcelona, Diagonal 643, Barcelona, 08028 Spain

**Keywords:** Embryology, Viral pathogenesis, Translational research

## Abstract

Zika virus (ZIKV) infection results in an increased risk of spontaneous abortion and poor intrauterine growth although the underlying mechanisms remain undetermined. Little is known about the impact of ZIKV infection during the earliest stages of pregnancy, at pre- and peri-implantation, because most current ZIKV pregnancy studies have focused on post-implantation stages. Here, we demonstrate that trophectoderm cells of pre-implantation human and mouse embryos can be infected with ZIKV, and propagate virus causing neural progenitor cell death. These findings are corroborated by the dose-dependent nature of ZIKV susceptibility of hESC-derived trophectoderm cells. Single blastocyst RNA-seq reveals key transcriptional changes upon ZIKV infection, including nervous system development, prior to commitment to the neural lineage. The pregnancy rate of mice is >50% lower in pre-implantation infection than infection at E4.5, demonstrating that pre-implantation ZIKV infection leads to miscarriage. Cumulatively, these data elucidate a previously unappreciated association of pre- and peri-implantation ZIKV infection and microcephaly.

## Abstract (web summary)

Here, using human cells and mouse models, the authors show that Zika virus can infect preimplantation trophectoderm. Pre-implantation infection can affect nervous system development and survival of neural progenitors, and can result in miscarriage or spontaneous abortion.

## Introduction

Perinatal infections, including Zika virus (ZIKV), account for 2−3% of all congenital abnormalities, and are a major cause of maternal and fetal illness^1^. The mechanisms underlying both mother-to-fetus viral transmission (vertical transmission) and the pathogenesis of congenital malformations and related miscarriages are still largely unknown. The emergence and rapid spread of ZIKV that began in 2014 has emphasized the importance of understanding congenital viral infections. ZIKV is a single-stranded positive-sense RNA virus of the *Flaviviridae* family that is transmitted by mosquitoes, as well as vertically from mother to fetus, sexually, and through blood transfusions. Several studies have highlighted that ZIKV can be detected in multiple types of maternal and fetal tissues, including the placenta, amniotic fluid, and fetal brains with microcephaly^2,3^.

Several studies have been performed to examine the role of placental cells in mother-to-fetus vertical transmission (Supplementary Tables 1 and 2). Using mid-^4^ and late-gestation placentas^5^ and organ culture^6^, or explants from first-trimester chorionic villi, ZIKV has been shown to infect primary human placental cells and explants, including cytotrophoblasts, endothelial cells, fibroblasts, and Hofbauer cells^7–12^. However, the role of human trophoblast cells during ZIKV infection has been controversial. Trophoblast cell lines, such as BeWo^13^, JEG3^14,15^, JAR^16^, HTR8/SVneo^17,18^, Sw.71 cells^19^, and human placenta cell lines^20^ are permissive to viral infections. However, human trophoblasts from mid-gestation^21^ and full-term^17^ placentas are refractory to ZIKV infection through the release of paracrine effectors, including the constitutive release of type III IFNs. Trophoblasts, including cytotrophoblasts and syncytiotrophoblasts, were derived from human embryonic stem cells (hESCs), and are permissive to ZIKV infection^22–24^.

ZIKV infection has been associated with adverse pregnancy outcomes, intrauterine growth restriction (IUGR), fetal developmental abnormalities, microcephaly, and fetal demise^3^. Notably, an increased risk for adverse outcomes and severe abnormalities has been linked to the timing of infection during gestation^25^. For example, Brasil et al.^25^ reported that 55% of pregnancies resulted in adverse outcomes when the mother was infected during the first trimester, whereas 52 and 29% resulted in adverse outcomes when infected in the second and third trimesters, respectively. Indeed, several studies have shown that the cells and tissues isolated from early gestation are more susceptible to ZIKV infection, including, but not limited to, isolated first trimester trophoblast cells, Hofbauer cells, amniotic cells, and placental explants^5,12,17,24,26–29^. Furthermore, a panel of animal studies in monkey and mouse has demonstrated a time-dependent effect of ZIKV infection on maternal and fetal health^14,26,30^ (Supplementary Table 2). An early study by Miner et al.^14^ reported that maternal infection of E6.5 and E7.5 pregnant *Ifnar1*^−/−^ mice, or C57BL/6 mice treated with IFNAR blocking antibody, resulted in fetal demise and intrauterine growth restriction, respectively. Jagger et al.^4^ demonstrated that C57BL/6 mice pre-treated with IFNAR blocking antibody infected at E6 resulted in fetal demise, whereas infection at E12 had no fetal effects. They further established higher placental vRNA levels present when infected at E6 as compared to later stage infection^4^. Alternative routes of infection also demonstrated a stage-dependent effect. One study directly inoculated the uterine walls of CD1 mice and found that ZIKV infection at E10, but not E14, reduces fetal viability^27^. Another study infected C57BL/6NCrl and interferon deficient (*Irf3*^−/−^*Irf7*^−/−^or *Ifnar1*^*+/-*^) mice intravaginally, mimicking ZIKV sexual transmission, and found reduced fetal weights^31^. Chen et al.^32^ examined ZIKV infection of *Ifnar*^−/−^ dams at E5.5 (early), E10.5 (mid-), and E15.5 (late) and found that infection at early gestation led to complete fetal demise, while infection at mid-gestation resulted in IUGR and partial fetal demise, and infection during late gestation resulted in no overt fetal abnormalities^32^. Finally, rhesus monkeys infected early in pregnancy (weeks 6–7 of gestation) exhibited prolonged maternal viremia and fetal neuropathology^30^.

To date, however, little is known about ZIKV infection during the earliest stages of pregnancy, at pre- and peri-implantation. Here, we demonstrate that pre-implantation human and mouse embryos can be productively infected with ZIKV. We examine the effect of vertical ZIKV transmission during the earliest stages of development using ex vivo mouse and human blastocyst cultures, hESC-derived trophectoderm cells, and in vivo in the mouse. Hence, our results show that pre- and peri-implantation ZIKV infection causes miscarriage via trophectoderm infection in a large percentage of embryos, and in surviving fetuses this early infection impairs fetal development and causes neural progenitor cell death. This study provides insights into early ZIKV infection and suggests a previously unexpected association of pre- and peri-implantation ZIKV infection and microcephaly.

## Results

### ZIKV susceptibility of mouse and human trophectoderm

**Fig. 1:**
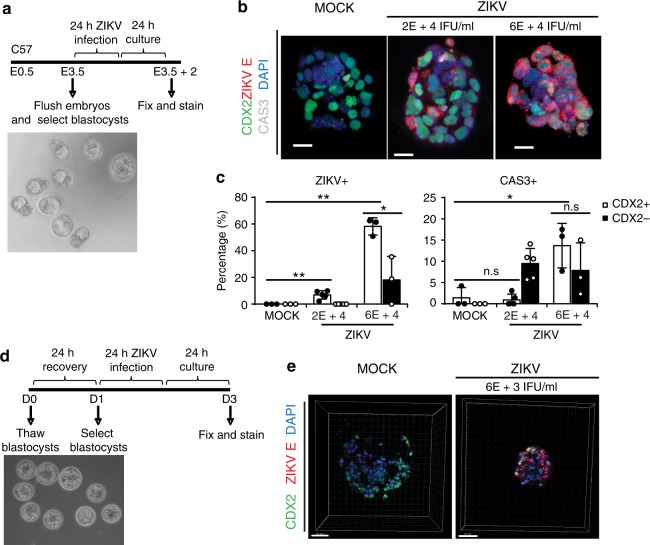
Human and mouse trophectoderm can be infected with ZIKV. **a** Scheme of ZIKV infection on mouse blastocysts in vitro, including phase contrast image of blastocyst-staged embryos prior to infection. **b** ZIKV infection (ZIKV E) and cell apoptosis (Cleaved Caspase3/CAS3) in mouse trophectoderm (CDX2) was indicated by immunostaining of mouse blastocysts at 24 h post infection with MR766 strain ZIKV at 2 × 10^4^ IFU ml^−1^ and 6 × 10^4^ IFU ml^−1^. *n* = 3 MOCK, *n* = 5 ZIKV 2 × 10^4^ IFU ml^−1^, *n* = 3 ZIKV 6 × 10^4^ IFU ml^−1^. Scale bar = 20 µm. **c** Quantification of ZIKV E^+^ cells and CAS3^+^ cells among CDX2^+^trophectoderm in embryos immunostained in **b**. *n* = 3 MOCK, *n* = 5 ZIKV 2 × 10^4^ IFU ml^−1^, *n* = 3 ZIKV 6 × 10^4^ IFU ml^−1^. **d** Scheme of ZIKV infection on human pre-implantation embryos in vitro, including phase contrast image of embryos prior to infection. **e** ZIKV (ZIKV E) infection in human trophectoderm (CDX2) was indicated by immunostaining of human embryos at 24 h post infection with MR766 strain ZIKV. *n* = 3 MOCK, *n* = 2 ZIKV. Scale bar = 50 µm. Data are presented as mean ± standard deviation. *P* values were calculated by multiple unpaired two-tailed Student’s *t-*test. *P < 0.05, **P < 0.01, *n.s.* – not significant. Source data for 1c are provided as a Source Data file

To study the effect of early ZIKV infection during pre- and peri-implantation, mouse blastocysts were infected ex vivo. Embryos were isolated from C57BL/6 mice at embryonic day 3.5 (E3.5), and those that developed to the blastocyst-stage (presence of blastocoel) were selected (Fig. 1a). Blastocysts were infected with MOCK or ZIKV at high and low doses (MR766 strain, 6 × 10^4^ IFU ml^−1^, and 2 × 10^4^ IFU ml^−1^) in hanging drop cultures for 24 h, and then transferred to fresh medium for another 24 h (Fig. 1a). At 24 h post-infection (hpi), the blastocysts were stained with antibodies against CDX2 (trophectoderm)^33^, ZIKV E antigen, and Cleaved Caspase 3 (CAS3, cell apoptosis marker), and evaluated using confocal microscopy (Fig. 1b, Supplementary Movies 1–3). Mock infected blastocysts did not exhibit ZIKV^+^ cells (Fig. 1c left; *n* = 3 MOCK, *n*= 5 ZIKV 2 × 10^4^ IFU ml^−1^, *n* = 3 ZIKV 6 × 10^4^ IFU ml^−1^, 47–119 cells per embryo). Blastocysts infected with 2 × 10^4^ IFU ml^−1^ ZIKV revealed that 6.3 ± 0.03% of CDX2^+^ cells stained positive with ZIKV E antibody, while 0 ± 0% of CDX2^−^cells (epiblast and primitive endoderm cells) were ZIKV E^+^. For the blastocysts infected with 6 × 10^4^ IFU ml^−1^ ZIKV, 58.2 ± 0.06% of the CDX2^+^ cells and 18.0 ± 0.18% of the CDX2^−^ cells were ZIKV E-positive (Fig. 1c left). These data demonstrate that ZIKV can infect the trophectoderm (TE) of mouse blastocysts and the progenitor cells of all epithelial components of the placenta. In addition, to further characterize ZIKV infection, apoptosis was examined with cleaved CAS3. For the blastocysts infected with 2 × 10^4^ IFU ml^−1^ ZIKV, 0.4 ± 0.01% of CDX2^+^ cells were CAS3^+^, while 9.1 ± 0.04% of CDX2^−^ cells were CAS3^+^. For the blastocysts infected with 6 × 10^4^ IFU ml^−1^ ZIKV, 13.7 ± 0.05% of CDX2^+^ cells were CAS3^+^, while 7.8 ± 0.07% of CDX2^−^ cells were CAS3^+^ (Fig. 1c right). Thus, we demonstrate that ZIKV infection of pre-implantation embryos targets trophectoderm cells, is dose-dependent, and induces apoptosis.

We next performed ex vivo ZIKV infection of pre-implantation human embryos. Human embryos were thawed, and re-expanded for 4–24 h. Embryos were then infected with 6 × 10^3^ IFU ml^−1^ ZIKV (Fig. 1d), a viral titer several orders of magnitude lower than titers used in previous studies (5 × 10^5^ FFU ml^−1^ to 6 × 10^10^ RNA copies ml^−1^, Supplementary Table 2). Consistent with our data demonstrating ZIKV infection of mouse trophectoderm, ZIKV E antigen was detected in CDX2^+^ human trophectoderm (Fig. 1e).

### Dysregulated genes in blastocysts upon ZIKV infection

**Fig. 2:**
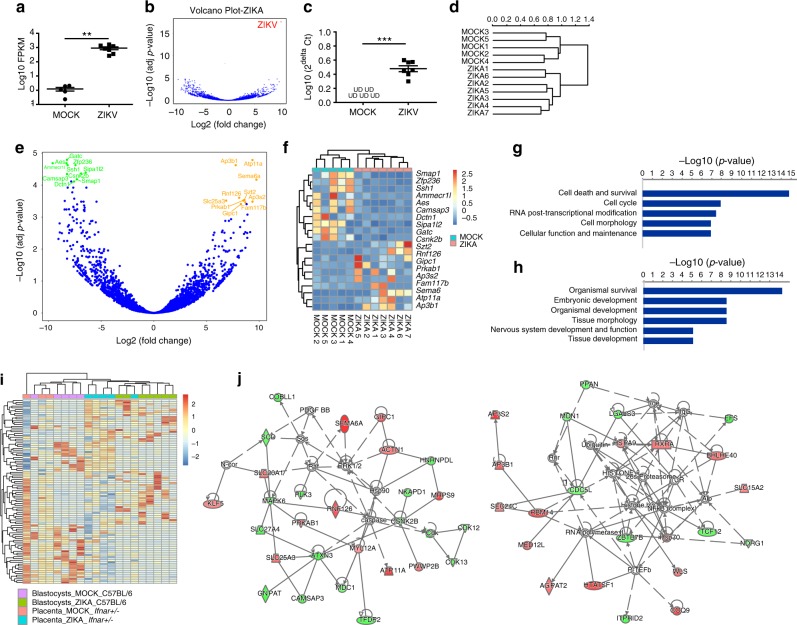
Single-blastocyst RNA-seq analysis of ZIKV-infected mouse blastocysts. **a** FPKM value of ZIKV vRNA in Mock or ZIKV-infected mouse blastocysts. *n* = 5 for MOCK, *n* = 7 for ZIKV. **b** Volcano plot of total RNA (including both mRNA and vRNA) of Mock or ZIKV-infected mouse blastocysts. ZIKV vRNA indicated in red. **c** qRT-PCR analysis of ZIKV vRNA in Mock or ZIKV-infected mouse blastocysts. *n* = 5 for MOCK, *n* = 7 for ZIKV. **d** Clustering analysis of Mock or ZIKV-infected mouse blastocysts. **e** Volcano plot of total mRNA of Mock and ZIKV-infected mouse blastocysts. Top 10 upregulated genes in ZIKV-infected blastocysts are highlighted in orange color. Top 10 downregulated genes in ZIKV-infected blastocysts are highlighted in green color. *p*_adj_ < 0.05, calculated by Wald test for differential expression with Benamini and Hochberg adjustment for multiple comparisons. **f** Heatmap of top 10 upregulated and downregulated genes in ZIKV versus mock-infected mouse blastocysts. Upregulated genes are demarked by red color and downregulated genes are demarked by blue color. **g**, **h** Ingenuity Pathway Analysis of cellular and molecular (**g**) and physiological (**h**) pathways that are significantly changed in ZIKV versus mock-infected mouse blastocysts. **i** Heatmap and clustering analysis (Top 50 upregulated and downregulated genes) of MOCK versus ZIKV-infected mouse blastocysts with MOCK versus ZIKV-infected mouse placentas (*Ifnar*^*+/−*^) in vivo. Color scale is set up with red as upregulation and blue as downregulation. **j** Ingenuity Pathway Analysis (IPA) of Top 50 upregulated and downregulated genes, which were analyzed in **i**. Upregulated genes are demarked by red color and downregulated genes are demarked by blue color. UD – undetectable. Data are presented as mean ± standard deviation. *P* values were calculated by unpaired two-tailed Student’s *t-*test. **P < 0.01, and ***P < 0.001. Source data for 2a,c are provided as a Source Data file

To determine the global transcriptional changes induced by ZIKV infection in pre-implantation embryos, RNA sequencing was performed on MOCK and ZIKV-infected mouse blastocysts. C57BL/6 blastocysts were isolated and infected as above (Fig. 1a), washed, RNA was then isolated and cDNA libraries were generated adapting a published protocol for low RNA samples^34^. RNA sequencing (Fig. 2a, b) and qRT-PCR (Fig. 2c) validated the presence of ZIKV vRNA in ZIKV-infected mouse blastocysts. Clustering analysis (Fig. 2d) showed that MOCK-infected and ZIKV-infected blastocysts are clustered separately. Ninety-six genes are upregulated and 167 genes are downregulated in ZIKV-infected blastocysts (Wald test for differential expression in DESeq2 package^35^ with Benamini and Hochberg adjustment for multiple comparisons, *p*_adj_ < 0.05, Fig. 2e). The top 10 upregulated and downregulated genes were presented as heatmap (Fig. 2f). Among the top upregulated genes are host genes that have been shown to be involved in virus life cycles. For example, *Ap3b1* encodes a clathrin-associated protein complex, which is required for HIV-1 release^36^. *Gipc1* was shown to directly interact with hepatitis B virus core protein in a human liver cDNA library screen^37^. Several top downregulated genes are involved in actin and microtubule dynamics, including *Ssh1*, *Camsap3*, and *Dctn1*. Flaviviruses, including Dengue Virus, have been shown to perturb these processes to facilitate replication and dampen immune responses^38^. In particular, viral penetration-induced calcium release facilitated HSV-1 intracellular trafficking through activating slingshot 1 (Ssh1), a phosphatase regulating actin filament dynamics^39^. HSV-1 induced the inactivation of cofilin by the downregulation of Ssh1, which together benefited viral replication^40^. Camsap3 has a strong inhibitory effect on Nef activity, a HIV-1-encoded protein^41^. Finally, Ingenuity Pathway Analysis revealed the top cellular and molecular functions altered after ZIKV infection include cell death and survival, cell cycle, as well as cellular function and maintenance (Fig. 2g). The top altered physiological functions include organismal survival and development and embryonic development (Fig. 2h). Notably, nervous system development and function was also affected after ZIKV infection in blastocyst-staged embryos. Collectively, these results suggest that pre-implantation ZIKV infection results in global transcriptional changes related to cell survival and embryonic development.

Gene expression profiles of MOCK and ZIKV-infected mouse blastocysts were also compared with mouse placentas (*Ifnar*^*+/*−^) infected at E5.5 and harvested at E10.5 (GSE98423, Supplementary Table 4, Fig. 2i)^42^. Clustering analysis indicated ZIKV-infected mouse blastocysts were clustered with ZIKV-infected mouse placentas, while MOCK blastocysts were clustered with MOCK placentas, which reflected the common reactions of gene expression induced with ZIKV infection. IPA analysis suggested two gene networks were most strongely disturbed by ZIKV infection: lipid metabolism (Fig. 2j, left) and cell mediated immune response (Fig. 2j, right).

### Dose-dependent effects of ZIKV infection in trophoectoderm

Although experiments using human embryos show direct evidence of ZIKV infection in human TE cells (Fig. 1d, e), it is technically and ethically challenging to perform large-scale studies using human embryos. Previous studies have reported strategies to differentiate human embryonic stem cells (hESCs) into the TE lineage^43–45^. Here, HUES8 hESCs were cultured in mouse embryonic fibroblast conditioned medium supplemented with 100 ng ml^−1^ BMP4 for 9 days (Supplementary Fig. 1a). Cells at day 4 and day 9 post-differentiation were examined. By immunocytochemistry analysis, at day 4, more than 90% of the cells express early trophoblast markers KRT7 and CDX2 (Supplementary Fig. 1b left, c). At day 9, hCG2 expression emerged (Supplementary Fig. 1b right). RNA-sequencing analysis suggested that day 4 cells were clustered together with TE isolated from human blastocysts, while hESCs were clustered together with epiblasts of human blastocysts (GSE36552 and GSE66507, Supplementary Table 4, Supplementary Fig. 1d)^46,47^. GSEA analysis further confirmed the TE gene set (genes that are 4 fold upregulated in TE vs epiblast) is enriched in day 4 cells (Supplementary Fig. 1e), which further validates the TE identity of day 4 cells. Based on these results, we have designated day 4 cells as trophectoderm cells (TECs). qRT-PCR confirms the expression of TE markers in day 4 TE cells, including *GATA3, EOMES*, and *ELF5*, and the upregulation of mature trophoblast markers in day 9 cells, including *CGA, CGB, GCM1, and HLA-G* (Supplementary Fig. 1f). In addition, ELISA results showed that day 9 cells, but not day 4 cells, secrete human chorionic gonadotropin (hCG, Supplementary Fig. 1g), the key pregnancy hormone secreted by mature synctiotrophoblast cells that plays several essential functional roles to maintain pregnancy^48^.

**Fig. 3:**
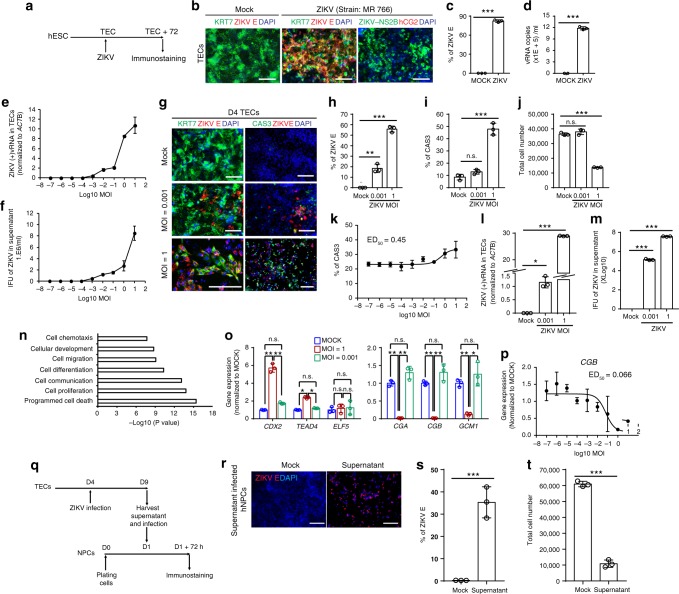
Distinct outcomes of dose-dependent ZIKV infection on hESC-derived TECs. **a** Scheme of ZIKV infection on TECs. **b**, **c** Immunostaining of ZIKV E, ZIKV NS2B, KRT7, and hCG in hESCs-derived TECs at 72 h post-infection of MR766 strain ZIKV (MOI = 0.25, Scale bar = 100 µm). Percentage of ZIKV E^+^ cells was quantified in **c**. *n* = 3. **d** qRT-PCR quantification of ZIKV vRNA in supernatant at 72 hpi of MR766 strain. *n* = 3. **e**, **f** Dose curve of ZIKV infection on day 4 TECs with ZIKV (MR 766, MOI ranging from 10^−7^ to 10^1^). Intracellular level of ZIKV vRNA at 72 hpi was quantified with qRT-PCR (**e**); while infectious virus in the supernatant 72 hpi was quantified using Vero assay (**f**), respectively. *n* = 3. **g**–**j** Immunostaining of ZIKV E and CAS3 in TECs at 72 hpi with high (MOI = 1) and low dose (MOI = 0.001) of MR766 strain ZIKV, Scale bar = 100 µm. The percentage of ZIKV E + cells was quantified in **h**. Percentage of apoptotic cells was quantified in **i**. Cell number was quantified in **j**. *n* = 3. **k** Immunostaining for CAS3 of TECs at 72 hpi with ZIKV (MR 766, MOI ranging from 10^−7^ to 10^1^). **l** qRT-PCR quantification of intracellular ZIKV ( + ) vRNA in TECs at 72 hpi with high (MOI = 1) and low dose (MOI = 0.001) of MR766 strain ZIKV, *n* = 3. **m** Vero assay to quantify ZIKV infectious particles in the supernatant at 72 hpi with high and low dose of MR766 strain ZIKV. *n* = 3. **n** Gene Ontology analysis of genes upregulated with ZIKV-infected vs. mock-infected TECs. **o** qRT-PCR analysis of TECs (left) and trophoblast (right) markers in ZIKV-infected vs. mock-infected TECs as described in **g**. *n* = 3. **p** qRT-PCR analysis of trophoblast marker, *CGB*, of TECs at 120 hpi with ZIKV (MR 766, MOI ranging from 10E-8 to 10). **q** Scheme of hNPCs infected with the supernatant harvested at120 hpi with low dose of MR766 strain ZIKV (MOI = 0.001). **r**–**t** Immunostaining of ZIKV E in NPCs infected with supernatant of TECs (1:2 dilution) schemed in **q**. Scale bar = 100 µm. Percentage of ZIKV E + cells was quantified in **s** and cell number was quantified in **t**. *n* = 3. Data are presented as mean ± standard deviation. *P* values were calculated by unpaired two-tailed Student’s *t*-test. *P < 0.05, **P < 0.01, and ***P < 0.001, *n.s.* – not significant. Related to Supplementary Fig. 1 and 2. Source data for 3c–f, h–m, o–p, s–t, are provided as a Source Data file

To determine whether hESC-derived TECs show similar susceptibility of ZIKV infection as human primary TE, TECs were infected with ZIKV (MR766 strain, MOI = 0.25; Fig. 3a). At 72hpi, ZIKV E antigen was detected in TECs (Fig. 3b, c). qRT-PCR further confirmed that ZIKV vRNA was present in the supernatant of TECs (Fig. 3d). Thus, these data demonstrate that hESC-derived TECs are susceptible to ZIKV infection, consistent with our finding that primary human TE cells can be infected with ZIKV. To explore whether the susceptibility of TECs to ZIKV is dose-dependent, TECs were infected with multiple doses of ZIKV (MR766 strain, MOI ranging from 10^−7^ to 10^1^). Intracellular level of ZIKV vRNA at 72 hpi was then measured with qRT-PCR (Fig. 3e). Infectivity of viral particles at 72 hpi in the supernatant was quantified using the Vero assay (Fig. 3f). Both assays indicated the TECs were infected by ZIKV and propagated ZIKV in a dose-dependent manner.

To further investigate TEC ZIKV infection, TECs were infected with two doses ZIKV (MOI = 1 and MOI = 0.001). MOI = 1 was chosen as the high dose used in many of the previous studies (Supplementary Table 1). MOI = 0.001 was the lowest dose that ZIKV vRNA and infectious viral particles can be detected at 72 hpi (Fig. 3e, f), and is considered the low dose. At both doses, ZIKV E protein in TECs can be detected by immunocytochemistry (Fig. 3g,h). High dose (MOI = 1), but not low dose (MOI = 0.001) of ZIKV infection, significantly induced cellular apoptosis as demonstrated by the increasing percentage of CAS3^+^ cells (Fig. 3i). Moreover, high dose (MOI = 1) ZIKV infection significantly decreases the total cell number (Fig. 3j). To further validate that ZIKV causes the cell apoptosis through a dose-dependent manner, we performed immunostaining for cell apoptosis marker, CAS3, at 72 hpi of TECs infected with a broad range of ZIKV (MR766 strain, MOI ranging from 10^−7^ to 10^1^). ZIKV infection increases the percentage of CAS3^+^ cells through a dose-dependent manner with ED_50_ = 0.45 (Fig. 3k). Moreover, ZIKV vRNA was detected in both high dose (MOI = 1) and low dose (MOI = 0.001) ZIKV-infected TECs (Fig. 3l). Furthermore, TECs infected by both high dose (MOI = 1) and low dose (MOI = 0.001) ZIKV propagated infectious virus as confirmed by Vero assay (Fig. 3m). Furthermore, the Asian strain of ZIKV (FSS13025) can also infect TECs (Supplementary Fig. 2a, b left) and induce cell apoptosis (Supplementary Fig. 2a, b right), suggesting this effect is not ZIKV strain dependent. RNA-seq was performed to compare the global transcriptional change between high dose (MOI = 1) ZIKV infected and Mock infected cells. Since nearly 90% cells express KRT7 and CDX2 (Supplementary Fig. 1b, c), the analysis of the entire population reflects the major changes in TECs. Gene ontology analysis suggested the upregulated categories in ZIKV-infected cells, which confirmed that cell death was induced by high dose of ZIKV infection (Fig. 3n), which is consistent with the conclusion from single-blastocyst RNA-seq analysis using mouse blastocysts.

To investigate how ZIKV infection influences trophoblast differentiation, qRT-PCR was used to compare the expression of TE markers *CDX2, TEAD4*, and *ELF5*, and trophoblast markers *CGA, CGB*, and *GCM1*, in TECs infected with MOCK, high (MOI = 1), or low dose (MOI = 0.001) of ZIKV. TECs infected with high dose ZIKV show increased expression of TEC markers and significantly decreased trophoblast markers (Fig. 3o), indicating that high-dose ZIKV infection disrupts TEC differentiation. Interestingly, TECs infected with low-dose ZIKV maintain survival (Fig. 3i, j) and the capacity to further differentiate to trophoblasts (Fig. 3o). To further validate the dose-dependent effect of ZIKV on TEC differentiation, TECs were infected with a broad range of ZIKV doses (MR766 strain, MOI ranging from 10^−7^ to 10^1^) and monitored for the expression of trophoblast marker, *CGB*, at 120 hpi. Indeed, ZIKV infection blocks TEC differentiation through a dose-dependent manner with IC_50_ = 0.066 (Fig. 3p). Staining with Ki67 antibody and EdU was used to monitor the proliferation of TEC at 72 hpi. Consistent with the result that ZIKV infection blocks TEC differentiation through a dose-dependent manner, ZIKV infection also affects cell proliferation through a dose-dependent manner. At high doses (MOI = 1 or 10), cells were maintained at the TEC stage, which show strong proliferation capacity (Supplementary Fig. 2c).

Since TECs infected by both high and low dose ZIKV can propagate infectious ZIKV particles (Fig. 3m), supernatant derived from TECs with low dose (MOI = 0.001) ZIKV infection was diluted 1:2 and applied to human neuronal progenitor cells (hNPCs) (Fig. 3q), the major cell type involved in ZIKV induced microcephaly. hNPCs can be effectively infected by the supernatant of TECs (Fig. 3r, s) and infection caused a decrease in the total cell number (Fig. 3t). Taken together, these data provide evidence that ZIKV infection of human TECs will lead to dramatic cell death, while low dose ZIKV infection propagates infectious virus that putatively transmits to fetal neuronal cells.

### Effects of murine pre- and peri-implantation ZIKV infection

To determine whether pre-and peri-implantation ZIKV infection results in pathological changes to the fetus in vivo, two mouse models deficient in type I IFN signaling were utilized.

First, C57BL/6 mice were mated and treated with anti-mouse IFNAR neutralizing antibody every other day beginning at E1.5, and infected subcutaneously with high dose ZIKV (MR766, 5 × 10^5^ IFU) or MOCK at E2.5 (Supplementary Fig. 3a). Females treated with IFNAR antibody alone were used as a control for any effects of the neutralization antibody. Only 57% (8/14) of ZIKV-infected females were pregnant at E6.5 compared to 89% (8/9) of MOCK infected controls and 83% (5/6) of IFNAR antibody treated controls (Supplementary Fig. 3b).

**Fig. 4:**
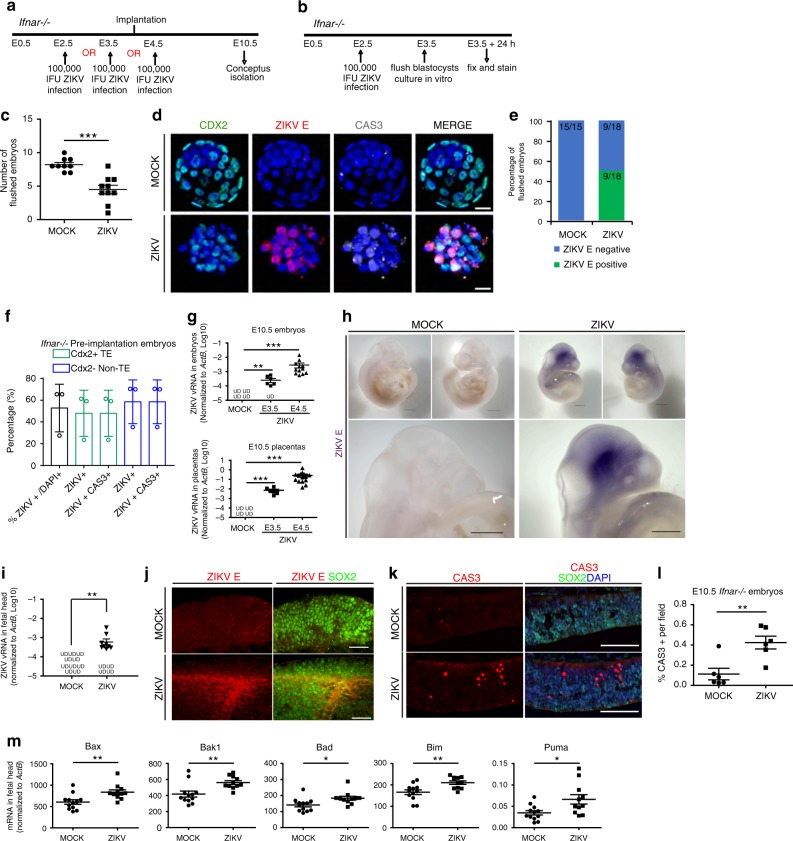
Effects of pre- and peri-implantation ZIKV infection on mouse embryos. **a** Scheme of ZIKV infection in pregnant *Ifnar*^−/−^ mice. **b** Scheme of ZIKV infection of mouse blastocysts in vivo and propagation in vitro. **c** Number of blastocysts recovered from pregnant *Ifnar*^−/−^ mice infected in **b**. *n* = 9 MOCK, *n* = 10 ZIKV. **d** Immunostaining of ZIKV E and CAS3 in blastocysts infected in **b**. Scale Bar = 20 µm. **e** Percentage of recovered blastocysts staining positive and negative for ZIKV E infected in **c**. *n* = 15 MOCK, *n* = 18 ZIKV. **f** Quantification of ZIKV E and CAS3 among CDX2^+^ and CDX2^-^ cells from blastocysts infected in **b**. *n* = 3. **g** Quantification of ZIKV vRNA level in E10.5 embryos (upper) and placentas (lower) infected at E3.5 and E4.5 in **a**. *n* = 4 for MOCK embryos and placentas, *n* = 7 for E3.5 embryos and placentas, and *n* = 12 for E4.5 embryos, *n* = 15 for E4.5 placentas. **h** Whole-mount chromogenic immunohistochemistry for ZIKV E antigen (purple precipitate) in E10.5 embryos of *Ifnar*^−/−^ dams infected at E4.5 in **a**. Scale bar = 500 µm. *n* = 3. **i** Quantification of ZIKV vRNA level in heads of E10.5 embryos infected in **a**. *n* = 10 for MOCK, *n* = 13 for ZIKV. **j** Co-staining of SOX2 with ZIKV E in E10.5 embryonic brains infected in **a**. Scale bar = 50 µm. **k** Co-staining of CAS3and SOX2 in E10.5 embryonic brains infected in **a**. Scale bar = 100 µm. **l** Quantification of CAS3 + area in **k**. *n* = 6 sections each. **m** Quantification of apoptotic genes expression in heads of E10.5 embryos infected in **a**. *n* = 12 for MOCK, *n* = 11 for ZIKV. UD – undetectable. Data are presented as mean ± standard deviation. *P* values were calculated by unpaired two-tailed Student’s *t-*test. *P < 0.05, **P < 0.01, and ***P < 0.001. Related to Supplementary Fig. 3. Source data for 4b, d, f–h, j, m, n are provided as a Source Data file

Moreover, we used *Ifnar*^−/−^ mice as a second mouse model. *Ifnar*^-/-^ mice were mated and infected subcutaneously with ZIKV (MR766, 1 × 10^5^ IFU) or MOCK at E2.5, E3.5, or E4.5 (Fig. 4a). The ZIKV viral titer chosen falls within the range used in previous studies (10^3^ FFU to 3.4 × 10^5^ PFU, Supplementary Table 2), and is much lower than the titer found in semen, testes, and blood of human, mouse, and non-human primates (Supplementary Table 3). Conceptuses were isolated, counted and processed for qRT-PCR and immunostaining at E10.5. Only 33% (1 of 3, E2.5 and 2 of 6, E3.5) of pre-implantation ZIKV-infected females were pregnant at E10.5, as compared to 100% (3 of 3, E2.5) and 80% (4 of 5, E3.5) of MOCK infected controls, respectively (Table 1). Interestingly, 88.89% (8 of 9) females infected at E4.5 were pregnant at E10.5 compared to 100% (3 of 3) of E4.5 MOCK infected controls (Table 1). These results indicate that pre-implantation ZIKV infection (E2.5 and E3.5) leads to miscarriage in the initial stages of pregnancy in a majority of embryos, potentially due to the in vivo ZIKV infection of the developing blastocysts.

**Table 1:** The pregnancy rate of infected *Ifnar*^−/−^ mice in Fig. 4a

E10.5 *Ifnar*−/−	E2.5		E3.5		E4.5	
	MOCK	ZIKV	MOCK	ZIKV	MOCK	ZIKV
# of pregnant	3	1	4	2	3	8
# of not pregnant	0	2	1	4	0	1
Total # of plugs	3	3	5	6	3	9
Pregnant/plugged ratio (%)	100	33.33	80	33.33	100	88.89

To verify if maternal ZIKV administration results in infection of blastocyst-staged embryos, *Ifnar*^−/−^ mice were mated and infected at E2.5 with ZIKV (MR766, 1 × 10^5^ IFU) or MOCK. Blastocysts were flushed at E3.5 and cultured in hanging drops for 24 h (Fig. 4b). Significantly fewer blastocysts were recovered from ZIKV-infected females as compared to the MOCK infected females (Fig. 4c), further substantiating our previous results that pre-implantation ZIKV infection leads to miscarriage (Table 1). After 24 h in culture, blastocysts were fixed and stained. ZIKV E and CAS3 were detected in CDX2^+^ TE cells of blastocysts (Fig. 4d). 50% (9 of 18) of blastocysts collected from ZIKV-infected females and 0% (0 of 15) from MOCK infected females were ZIKV E^+^ (Fig. 4e). Furthermore, the morphology of ZIKV E^+^ blastocysts demonstrated a loss of the distinct organization of the compact CDX2^−^ inner cell mass and the outer CDX2^+^ TE (Fig. 4d). Over 50% of the total cells in the blastocysts from ZIKV-infected females were ZIKV E^+^, consisting of both CDX2^+^ TE cells and CDX2^−^ cells (Fig. 4f). Every ZIKV E ^+^ cell was also CAS3^+^, demonstrating that pre-implantation ZIKV infection of the mother can productively infect the pre-implantation embryos and induce embryonic demise.

To determine the effect of pre- or peri-implantation ZIKV infection on embryonic development, *Ifnar*^−/−^ mice were infected at E3.5 or E4.5 and tissues were collected at E10.5 (Fig. 4a). ZIKV vRNA was then quantified in the maternal spleen and brain, as well as E10.5 placentas and embryos of infected female mice. ZIKV-infected mice showed ZIKV vRNA in maternal spleen and brain (Supplementary Fig. 3c), which validates the successful maternal infection. ZIKV vRNA was also detected in the placentas and embryos (Fig. 4g). Interestingly, higher doses of ZIKV vRNA were detected in the placentas and embryos of mice infected at E4.5 than the mice infected at E3.5.

Since ZIKV vRNA can be detected in E10.5 embryos of females infected at pre- and peri-implantation, whole mount chromogenic immunohistochemistry was performed to determine the localization of ZIKV infection (Fig. 4h). E10.5 embryos were isolated from MOCK and ZIKV-infected *Ifnar*^−/−^ females, fixed, and stained with an anti-ZIKV E antigen antibody. ZIKV E-positive staining was observed enriched in the heads of E10.5 embryos (purple precipitate) while MOCK infected embryos were ZIKV E^-^ (Fig. 4h). qRT-PCR further validates the presence of ZIKV vRNA in the head of E10.5 embryos (Fig. 4i). To determine the cell type susceptible to ZIKV infection, E10.5 embryos from *Ifnar*^−/−^ females were embedded in low melt agarose, thick vibratome sectioned, and co-stained for the neural progenitor marker, SOX2, and ZIKV E antigen. ZIKV E-positive cells were also SOX2 positive, indicating that ZIKV targets neural progenitor cells (Fig. 4j). Interestingly, ZIKV can target the developing brain, even when infected at the earliest stages of development, prior to the commitment of these cells to the neural lineage. Co-staining of SOX2 with CAS3 showed higher apoptosis in the neural progenitors of ZIKV-infected E10.5 embryos (Fig. 4k, l). This was further validated by qRT-PCR, which showed the upregulation of pro-apoptotic genes (Fig. 4m).

Collectively, these data demonstrate that ZIKV infection at pre- and peri-implantation can infect cells of the developing conceptus and can propagate virus over time, causing infection and cell death in neural progenitors. Furthermore, ZIKV infection during pre-implantation results in embryonic death and pregnancy loss.

## Discussion

We utilize in vitro and in vivo assays to demonstrate that pre-implantation ZIKV infection affects fetal development. Using both mouse and human pre-implantation embryos, we provide direct evidence that ZIKV can infect TE, induce global transcriptional changes, alter differentiation, and lead to CAS3-mediated cell death. Using hESC-derived TECs we demonstrate that ZIKV affects TECs’ survival and differentiation through a dose-dependent manner, in which high dose ZIKV infection of TECs leads to dramatic cell death, while TECs infected with low dose ZIKV propagate infectious virus that further infect fetal neuronal cells. Previous studies have shown that ZIKV can infect trophoblast cells and that cells from the first trimester are more susceptible to ZIKV infection, however most of these studies examined tissues or cells later than 7 weeks gestation in the human^8,11,14,17,19,21^ (Supplementary Table 1). Our study shows that ZIKV can infect TE cells, which arise ~5–12 days after fertilization, at pre- and peri-implantation stages of trophoblast development.

Previously published studies have demonstrated that ZIKV can replicate in term placentas and late stage placental trophoblasts^4,8^ (Supplementary Table 1). Our data focuses on trophoblasts from the earliest stages of development using mouse and human pre-implantation embryos and hESC-derived TECs in vitro. Previous work has demonstrated that hESC-derived trophoblasts can become infected by ZIKV. One study revealed that these cells are productively infected and undergo lysis after infection with ZIKV strain MR766, but not FSS13025^24^; whereas our results show ZIKV can induce cell apoptosis without ZIKV strain differences (Fig. 3g, Supplementary Fig. 2a, b). One possible reason for this is that different differentiation protocols were used and that different stages of the differentiated cells were analyzed in the two studies. Sheridan et al.^24^ used BMP4/A83–01/PD173074 to induce differentiation. Based on the description, day 8 cells used in Sheridan et al.^24^ represent syncytiotrophoblast cells^23^. In contrast, our study used BMP4 alone. We performed extensive analysis to confirm that our day 4 cells represent very early stage TECs by immunostaining, qRT-PCR, and RNA-seq (Supplementary Fig. 1). Importantly, the cells used by Sheridan et al.^24^ have little to no expression of the key early TE markers CDX2, ELF5, EOMES, and ASCL1, suggesting that their cells have differentiated beyond TECs. Furthermore, Sheridan et al.^24^ only studied the infection and toxicity effect of ZIKV on trophoblasts and syncytiotrophoblasts. We not only detect ZIKV infection and cytotoxicity effects, but also find ZIKV infection affects differentiation of TECs through a dose-dependent manner. Another study by Aagaard et al. revealed conditions in which ZIKV infection in trophoblast cells does not affect their differentiation^8^. Different conclusions were generated due to the different starting population and ZIKV dose ranges. First, Aagaard et al. isolated and cultured trophoblasts from human placentas >7 weeks, whereas our study uses hESC-derived TECs (equivalent to 5–12 days after fertilization). In addition, we perform a more extensive dose curve and confirm that ZIKV infection affects differentiation of TECs through a dose-dependent manner^8^. Aagaard et al. only used one dose, 1 × 10^5^ vRNA copies, of ZIKV FLR strain, which is a similar condition as our low dosage of ZIKV infection (MOI = 0.001, MR766) that does not affect differentiation of hESC-derived TECs^8^. We find that ZIKV can infect TECs (Fig. 3e, f), cause apoptosis (Fig. 3k) and impair trophoblast differentiation (Fig. 3p) through dose-dependent manners. There are additional factors that might also impact the results of miscarriage versus CZS affected fetuses, such as timing of infection. The pregnancy/plugged ratio of E2.5 and E3.5 infection is only 33.3%; in contrast, the pregnancy/plugged ratio of E4.5 infection is 88.9% (Table 1), suggesting that the time of infection also affects results.

ZIKV has been reported to cause pregnancy loss in most primate species ^26,49,50^. These studies and others, however, have focused solely on post-implantation ZIKV infection, as summarized in Supplementary Table 2. Our studies demonstrate that pre-implantation ZIKV infection of trophectoderm leads to miscarriage or spontaneous abortion. Moreover, we find that at pre- and peri-implantation, ZIKV infects trophectoderm cells that then propagate the virus over time causing cell death in neural progenitors. In most previously published studies analyzing ZIKV in a time dependent manner in the mouse, embroys were infected at post-implantation stages, with E6.5 as the most evaluated time point during development^4,14,51–53^ (Supplementary Table 2). Recent studies demonstrated that ZIKV infection at E5.5^32,42,54^ or vaginally at E4.5^31^ resulted in spontaneously aborted fetuses. Limited studies, however, have looked at vertical transmission during pre- and peri-implantation, a critical time point in the development of the embryo. Our studies show that pre-implantation ZIKV infection of TE leads to miscarriage or spontaneous abortion. We examined pre- and peri-implantation vertical ZIKV transmission from mother to fetus, and demonstrate that the infection has time dependent effects. Pre-implantation ZIKV infection targets the developing embryo and induces miscarriage, whereas post-implantation ZIKV infection causes increased cell death in neural progenitors at E10.5, as a potential cause of microcephaly. In addition, in the mid-gestation embryo, ZIKV infection is highly restricted to the head, even under conditions where ZIKV infection occurs prior to commitment of the inner cell mass cells to the neural lineage. Indeed, unexpected expression changes in genes important for nervous system development are perturbed after ZIKV infection at E3.5, prior to neural cell commitment. Our study also describes global transcriptional changes that occur at the earliest stages of development after infection with ZIKV. Pre-implantation ZIKV infection also perturbs pathways important for cell survival and for embryonic development.

Furthermore, this study demonstrates that subcutaneous ZIKV injection of the mother at peri-implantation can not only infect the developing embryo, but can hone to the developing brain. We cannot directly determine the kinetics of when the virus reaches the uterine lumen to the exact time during development that the embryo is exposed to the virus. However, when infected at E2.5 and isolated at E3.5, we already see a reduction in the number of surviving embryos and the remaining embryos are highly ZIKV E^+^, suggesting that the kinetics of infection occurs rapidly in this system.

Together, we use mouse and human blastocysts, hESC-derived TECs, and mouse models to show that pre- and peri-implantation ZIKV infection is not limited to causing fetal demise. More importantly, pre- and peri-implantation ZIKV infection leads to increased death of neural progenitor cells in later embryos, which is the major cause of microcephaly. This highlights the importance of studying the role of infection during pre- and peri-implantation in virus-associated fetal defects.

## Methods

### ZIKV infection of mouse pre-implantation embryos ex vivo

Blastocyst-stage embryos were isolated from C57BL/6 females by flushing uteri with M2 Medium (Sigma) at E3.5. Embryos were washed with M2 medium, and zona pellucidas were removed with EmbryoMax Acidic Tyrode’s solution (EMD Millipore). After 4 h of recovery in DMEM/F12 supplemented with 10% serum at 37 °C and 5% CO2, embryos were infected with ZIKV (MR766 strain, diluted to 6 × 10^4^ IFU ml^−1^ and 2 × 10^4^ IFU ml^−1^) in 40 µl hanging drop cultures for 24 h. Embryos were then washed and transferred to hanging drops free of virus for another 24 h. Blastocysts were then processed for immunostaining. MOCK infected embryos were cultured in hanging drops for 48 h and processed as above.

Blastocyst-staged embryos were fixed with 4% paraformaldehyde for 30 min at room temperature. Embryos were permeabilized with 0.25% Triton-X/PBS, blocked with 2.5% donkey serum/0.25% TritonX/0.05% Saponin/PBS, and incubated with primary antibodies: CDX2, (Biogenex #AM392–5M, 25 mg ml^−1^), ZIKV E antigen (Biovision #A1102, 1:1000), Cleaved Caspase 3 (Cell Signaling # 9661L, 1:1000), overnight at 4° in blocking buffer. Blastocysts were incubated with secondary antibodies: Alexa488-donkey-anti-mouse (Jackson Immunoresearch, 1.5 µg ml^−1^), Alexa647-donkey-anti-rabbit (Jackson Immunoresearch, 0.75 µg ml^−1^), Alexa594-donkey-anti-human (Jackson Immunoresearch, 1:500), and stained with DAPI (1:1000).

Blastocysts were imaged using glass bottom dishes (Matek) on a Zeiss LSM 880 confocal microscope. To quantify the percentages of ZIKV^+^ and CAS^+^ TE and non-TE cells in C57 blastocysts infected ex vivo, confocal z-stack images were taken through the entire pre-implantation embryo. Cells were quantified using ImageJ. Due to the effect of the infection, the total number of DAPI^+^ cells varied among embryos. The total number of cells per embryo ranged from 47 to 119, with the high dose ZIKV samples having fewer total cells and the MOCK infected having higher total cells. Mean values were therefore expressed as percentages.

### ZIKV infection of human pre-implantation embryos ex vivo

Embryo donation for research was approved by Western Institutional Review Board (WIRB). Human blastocysts were donated for research as surplus after in vitro fertilization treatment. All participants provided written informed consent prior to their participation in this study. The embryo donors no longer wished to store their embryos for their own future reproductive use and consented to give their embryos over for broad research purposes, with the only requirement being that the research meets medical and ethical standards. The acquisition and use of these specific embryos was approved by WIRB. All experimental procedures follow ISSCR guideline. Embryos were scored according to Stephenson et al.^55^ as blastocysts from days 4–5. Frozen blastocysts were thawed using a commercially available thawing media (Quinn’s Advanced Thaw Kit, Origio). All blastocysts were cultured for 8–24 h in 20 μl drops of Global Total media (Life Global) under mineral oil at 37 °C in an atmosphere of 5% CO2 in air for recovery before Zika virus infection. Zona pellucidas were removed with EmbryoMax Acidic Tyrode’s solution (EMD Millipore) and subsequently washed in Global Total media (Life Global). After recovery for an additional 4 h, ZIKV infection (MR766 strain at 6 × 10^3^ IFU ml^−1^) was performed as above. Embryos were fixed in 4% paraformaldehyde and processed for immunostaining. Pre-implantation human embryos were immunostained and imaged as above, with the following modifications: primary antibodies (CDX2, Biogenex #AM392–5M, 25 mg ml^−1^; ZIKV E antigen (Absolute Antibody, D1–4G2–4–15, 1:100).

### hESC culture and generation of hESC-derived TECs

hESCs were grown on Matrigel-coated plates with mTeSR1 medium (Stem Cell Technology). Cells were maintained at 37 °C with 5% CO2. HUES8 cells were kindly provided by Harvard Stem Cell Institute. To induce TE differentiation, HUES8 were passaged using Accutase (STEM CELL) and replated at the density of 200,000 cell/well on matrigel-coated 6-well plates in mTeSR1 medium, which was referred as day 0. The next day, the cells were changed to MEF conditional medium (MEF-CM) with 4 ng ml^−1^ recombinant basic fibroblast growth factor (bFGF) (Invitrogen). The following days, the cells then cultured in MEF-CM supplemented with 100 ng ml^−1^ human BMP4 (StemRD) for the indicated number of days. MEF-CM was prepared by collecting cell supernatant from mouse Feeder Cells (MIT-global stem) cultured in hESC medium.

### ZIKA virus infection of hESC-derived TECs

Vero cell line and Mosquito cells were maintained in DMEM medium (Gibco) plus 10% heat-inactivated FBS with penicillin/streptomycin at 37 °C or 25°C respcetively. MR766 strain of ZIKV was obtained commercially (ZeptoMetrix) and then titrated by plaque assay in Vero cells. FSS13025 strain was propagated with Mosquito cells clone C6/36 (ATCC® CRL-1660™).

TECs derived from hESCs were infected with MR766 strain ZIKV at MOI = 1 (or other indicated dose) for 2 h at 37 °C. FSS13025 strain ZIKV was used at MOI = 10 (or indicated as other dose). Medium was refreshed after 2 h of infection and cells were then maintained for 3 days in culture.

### Immunocytochemistry

Cells were fixed with 4% PFA for 20 min at room temperature, blocked in Mg^2+^/Ca^2+^ free PBS containing 5% horse serum and 0.3% Triton-X for 1 h at room temperature, and then incubated with primary antibody at 4 °C overnight. The following primary antibodies have been used with given dilutions: mouse anti-Flavivirus group antigen antibody (ZIKV E) (1:2000; Millipore, clone D1-4G2-4-15), mouse anti-Ki67 (1:200, DAKO, MIB-1), rabbit anti-Ki67 (1:500, Thermofisher, SP-6), rabbit anti-cleaved caspase-3 (1:1000, Cell Signaling Technology, Asp15), mouse anti-CDX2 (1:100, BioGenex, MU392A-UC), and rabbit anti-KRT7 (1:500, Abcam, ab119697). The secondary antibodies include donkey anti-mouse, goat, rabbit or chicken secondary antibodies conjugated with Alexa-488, Alexa-594 or Alexa-647 fluorophore (1:500, Life Technologies). Nuclei were counterstained by DAPI. EdU incorporation was analyzed with Click-iT™ Plus Alexa Fluor™ 555 PicolylAzide Tool kit (Thermofisher, C10642).

### Quantification of ZIKV infectious in trophoblast culture

Vero cells were maintained with DMEM medium plus 10% fetal bovine serum and plated to 96-well plates (2 × 10^4^ cells/well) one night before infection. ZIKV containing supernatant was collected from the trophoblast culture and clarified with centrifugation (1200 rpm, 5 min at room temperature). Supernatant was then serial diluted ranging from 10 to 10^6^ fold to infect Vero cells for 2 h at 37 °C, and then replaced with semi-solid medium (alpha-MEM containing 10% fetal bovine serum and 1% methylcellulose). After 2 days of culture, cells were fixed with 4% PFA and stained with anti-ZIKV E antibody to highlight the localized cluster of infected cells.

### Re-infection of neural progenitor cells

hNPC 3113-3-21 line^56^ were maintained on growth factor reduced Matrigel (BD Biosciences) coated plates in NPC media and passaged at 1:3 ratio weekly with Accutase (Millipore). NPC media contains DMEM/F-12 Nutrient Mixture (ThermoFisher Scientific), 1xN2 (ThermoFisher Scientific), 1xB27 minus RA (ThermoFisher Scientific) and 20 ng ml^−1^ recombinant human bFGF (Peprotech).

NPCs were plated on Matrigel-coated 96-well plates the night before infection. Cells were infected with supernatant containing ZIKA virus at 37 °C for 2 h. 3 days after infection, cells were fixed and stained with anti-ZIKV E antibody.

### Single-blastocyst RNA sequencing

Blastocysts were isolated and infected as above. At 24hpi, embryos were washed with several drops of media and PBS. Blastocysts were processed for RNA extraction and cDNA libraries generation by adapting a protocol for low RNA samples^34^. Namely, single blastocysts were lysed in 5 µl lysis buffer containing an RT primer (TCGTCGGCAGCGTCAGATGTGNNNNNN). Samples were then reverse transcribed in a RT reaction containing a template-switching oligo (TCGTCGGCAGCGTCAGATGTGTATAAGAGACAGNNNNNNNNTATA(rG)(rG)(rG)). Pre-amplification PCR was performed using a PCR primer (TCGTCGGCAGCGTCAGATGTG). The concentration of the resulting cDNA libraries was measured with Qubit® 2.0 Fluorometer (ThermoFisher Scientific) and library preparation was performed with the Nextera XT Library Preparation Kit (Illumina, FC-131-1096), followed by library fragment size validation with BioAnalyzer (Agilent Technologies). Finally, 100 bp paired-end sequencing was completed on the Illumina HiSeq4000 platform at the Weill Cornell Genomics Resources Core Facility.

The sequencing reads were aligned to the mouse mm10 reference genome and the ZIKV genome (LC002520.1, strain: MR766) with STAR aligner at the same time^57^. Raw read counts were calculated using HTSeq-count^58^. Differential expression analysis was performed using DESeq2 package^35^. Samples were clustered based on expression of differentially expressed genes (adjusted *p*-value < 0.05) using hclust in R. The expression data for *Ifnar*^*+/−*^ placentas were retrieved from GEO (accession number: GSE98423, see Supplementary Table 4). Volcano plots were generated using R ggplot2 package, and top genes were highlighted in the plots using R ggrepel package. Pathway analysis was performed on differentially expressed genes (adjusted *P*-value < 0.1) using IPA (Ingenuity Pathway Analysis, QIAGEN Inc, https://www.qiagenbioinformatics.com/products/ingenuitypathway-analysis)^59^. The heat map plot and sample clustering was generated using R heatmap package.

### hESC-derived TEC RNA sequencing analysis

Mock- or ZIKV–infected (MR766 strain, MOI = 1) hESC-derived TECs were collected at 72 hpi for RNA-seq. Total RNA in each condition was extracted with Rneasy plus mini kit (Qiagen). The RNA was validated with a Bioanalyzer (Agilent Technologies) for quality, cDNA libraries preparation was done with TruSeq RNA Sample Preparation kit (Illumina), and then sequenced with the HiSeq4000 sequencer (Illumina) at Weill Cornell Genomics Resources Core Facility.

The single-end long generated reads were aligned to the human hg19 reference genome with Tophat2^60^. Raw read counts were calculated using HTSeq-count^58^. Differential expression analysis was performed using DESeq2 package^35^. The expression data for TE and EPI isolated from human blastocyst were retrieved from GEO (accession number: GSE36552 and GSE66507). The expression data were normalized per gene across samples by subtracting their mean and then dividing by their standard deviation. The normalization was performed separately for TE and EPI, and for hESC derived cells, to remove batch effects introduced by experiments. The heat map plot and sample clustering was generated using R “heatmap” package. Gene enrichment analysis was performed using GSEA software^61^.

### qRT-PCR

Total RNA of cultured cells and organs was extracted with Rneasy plus mini kit (Qiagen). Reverse transcription was carried out with High Capacity cDNA Reverse Transcription kit (ThermoFisher). For ZIKV vRNA, positive strand specific tagged primers were used. For ACTB, gene specific primer was used. For other genes, random hexamer primer was used.

For ZIKV vRNA and human ACTB or mouse Actb, qPCR reactions were performed with PrimeTime Gene Expression 2X Master Mix (IDTDNA) and probes for ZIKV and human or mouse ACTB. ZIKV expression level was then normalized to human ACTB or mouse Actb correspondingly. The sequences of primers/probes used in RT and qPCR are listed in Supplementary Tables 5 and 6.

### In vivo ZIKV infection

All animal protocols were approved and are in accordance with the Institutional Animal Care and Use Committee (IACUC) at Weill Cornell Medical College. C57BL/6 (Jackson Laboratories) mice were crossed and the date of visualization of the vaginal plug was termed E0.5. IFNAR neutralization antibody (MAR1-5A3, Leinco Technologies) was administrated to pregnant females with of 2 mg per mouse for each injection by intraperitoneal injectionat E1.5, E3.5, and E5.5. MR766 strain of virus was delivered to antibody treated mice with 5 × 10^5^ IFU at E2.5 by subcutaneous injection. Mice were euthanized by cervical dislocation at E6.5 and conceptuses were isolated and counted.

*Ifnar*^−/−^ mice (The Jackson Laboratory) were intercrossed and infected with ZIKV at E2.5, E3.5, or E4.5 (MR766 strain, 2 × 10^5^ IFU) via subcutaneous injection. Mice were euthanized by cervical dislocation and embryos were isolated at E10.5. Embryos were flash frozen and processed for RNA extraction or fixed in 4% paraformaldehyde for immunostaining.

### Immunohistochemistry on mouse tissues

E10.5 embryos were permeablized in TSP(PBS-0.5% TritonX-0.1% Saponin) overnight at 4 °C. Endogenous alkaline phosphatases were inactivated by incubation with Glycine-HCl, followed by blocking with 10% donkey serum in TSP overnight at 4 °C. Embryos were incubated with anti-ZIKV E primary antibody (Biovision #A1102, 1:750) overnight at 4 °C, followed by AP-donkey-anti-human secondary antibody (Jackson Immunoresearch, 1:1500) overnight at 4 °C. Embryos were activated in NTMT and AP staining was developed using NBT/BCIP (Roche) at 4 °C. Embryos were post-fixed in 4% PFA and imaged using a Nikon SMZ1500 dissection scope with a Nikon DS-FI3 camera.

For thick sections, E10.5 embryos were fixed in 4% paraformaldehyde (PFA), washed in PBS, and heads subsequently embedded in 4% low melting point agarose and sectioned at 150 µm thickness using a vibratome. Brain sections were permeabilized and blocked with 0.5% TritonX-100, 1% normal donkey serum, 1% DMSO, 20 µg ml^−1^ heparin, and 23 mg ml^−1^ glycine in phosphate-buffered saline (blocking buffer) overnight at room temperature, then at 37 °C for 3 h. Antibodies against SOX2 (Santa Cruz Y17) and ZIKV E (Biovision #A1102) were incubated at a concentration of 1:200 in blocking buffer at 37 °C for 6 h. After washing overnight in 0.5% TritonX-100 in phosphate-buffered saline (wash buffer) at room temperature, primary antibodies were detected using Alexa Fluor-conjugated secondary antibodies (Jackson ImmunoResearch) at a concentration of 1:250 diluted in blocking buffer for 2 h at 37 °C. After washing overnight at room temperature, tissues were post-fixed with ice cold 4% PFA at 4 °C for 5 min, then washed in PBS, optically cleared for imaging using the ClearT2 method^62^, and imaged using a Zeiss LSM 880 confocal microscope.

For thin sections, E10.5 embryos were fixed in 4% paraformaldehyde, cryopreserved in 30% sucrose, OCT embedded, and sectioned at 10 µm. Sections were blocked in 10% donkey serum in 0.1% TSP for 1 hour, stained for Cleaved Caspase 3 (Cell Signaling, 1:1000) and SOX2 (Santa Cruz Y17, 1:100) in 1% donkey serum-0.1% TSP overnight at 4 °C, followed by secondary antibody incubation in Alexa 594 donkey anti rabbit (1:500, Life Technologies) and Alexa 488 donkey anti goat (1:500, Life Technologies), and DAPI (Thermo Fisher, 1 μg ml^−1^), mounted in Prolong Gold (Thermo Fisher), and imaged on a Nikon Eclipse TE200 microscope with a mounted Hamamatsu C4742-95 camera.

### Statistical analysis

*n* = 3 independent biological replicates were used if not otherwise specifically indicated. *P* values were calculated by unpaired two-tailed Student’s *t*-test if not otherwise specifically indicated. *p < 0.05, **p < 0.01 and ***p < 0.001.n.s. indicates non-significant difference.

## Supplementary information

Supplementary Information

Description of Additional Supplementary Files

Supplementary Movie 1

Supplementary Movie 2

Supplementary Movie 3

Source Data

## Data Availability

All data supporting the findings in this study are available upon reasonable request. All RNA-sequencing datasets generated and used in this study are available from the NCBI Gene Expression Omnibus (GEO) portal, with the following GEO accession numbers: GSE133254 and GSE133396. The source data underlying Figs. 1c, 2a, c, 3c–f, h–m, o–p, s–t, 4b, d, f–h, j, m–n, and Supplementary Figs. 1c, 1f–g, 2b–c, 3b–c, are provided as a Source Data file. The raw data has been uploaded to: 10.6084/m9.figshare.9249065.
