# Supplementary material for: Pre- and peri-implantation Zika virus infection impairs fetal development by targeting trophectoderm cells
Source: Nat Commun. 2019 Sep 13;10:4155. doi: 10.1038/s41467-019-12063-2 (PMC6744420; doi:10.1038/s41467-019-12063-2)
Supplement: Supplementary file 1 — Supplementary Information [file 41467_2019_12063_MOESM1_ESM.pdf]

SUPPLEMENTARY INFORMATION.

Title: Pre- and Peri-implantation Zika Virus Infection Impairs Fetal Development by Targeting Trophectoderm Cells

First Authors: Tan, Lacko, Zhou et al.

Supplementary Figure 1

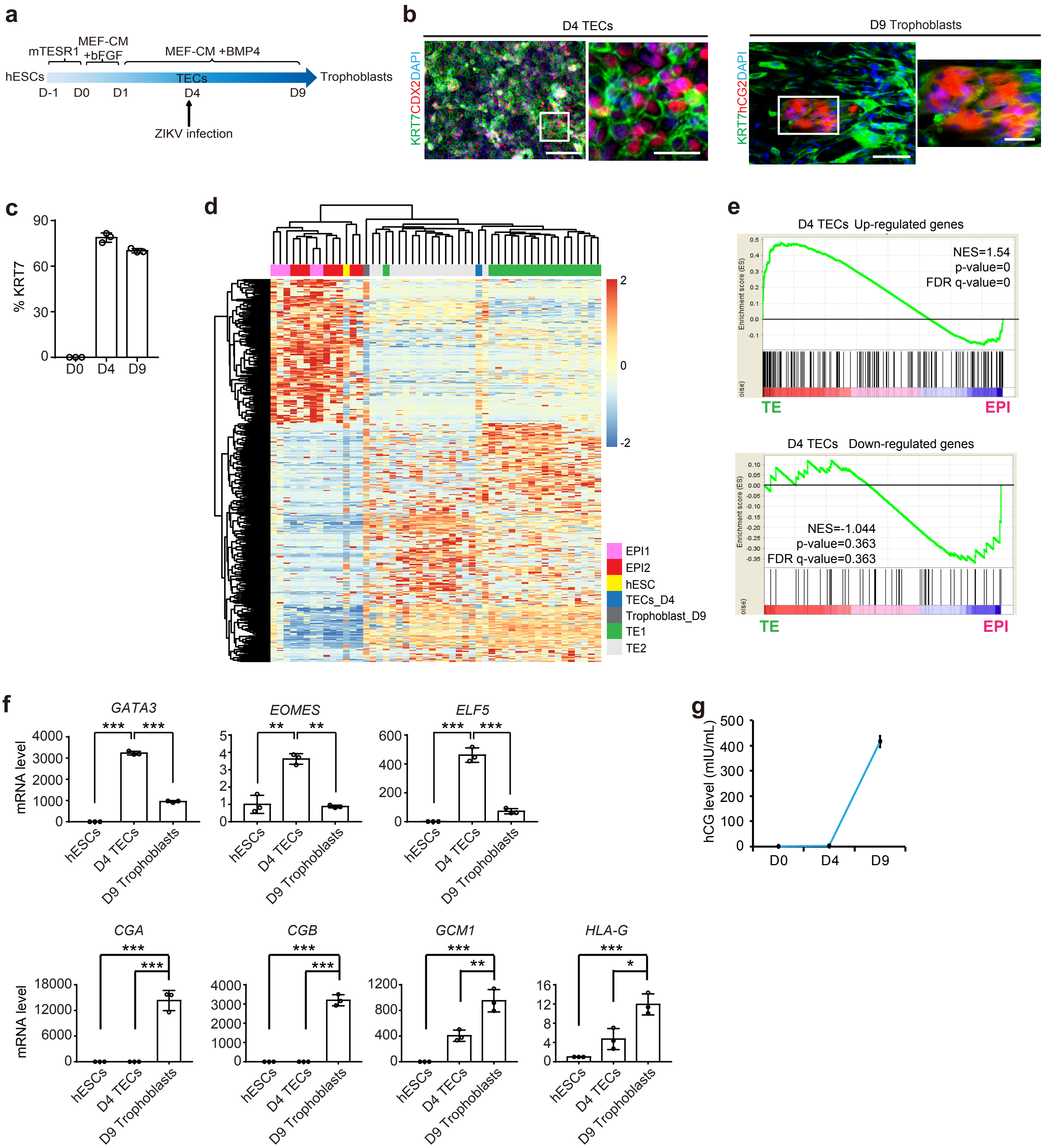

Supplementary Figure 1: Characterization of trophectoderm (TE) cells derived from hESCs. Related with Figure 2.

a, Scheme of TE cells generation from hESCs. **b,c,** Immunostaining of KRT7, CDX2 and hCG2 in hESC-derived TECs. **b**. In each panel, left photo is in lower magnification (Scale bar=100 µm), while right photo is higher magnification (Zoom image, scale bar=500 µm. Percentage of KRT7+ cells was quantified in **c**. N=3. **d,** Heatmap andclustering analysis of gene expression profile in hESC-derived TECs, comparing with trophectoderm and epiblast cells isolated from human blastocysts (Supplemental Table 4). Upregulated genes are demarked by red color and downregulated genes are demarked by blue color. **e,** GSEA analysis showed gene set upregulated in TECs vs. hESCs are highly enriched in trophectoderm isolated from human blastocysts (upper panel) and gene set downregulated in TECs vs. hESCs are highly enriched in epiblast cells isolated from human blastocysts (lower panel). **f,** qRT-PCR analysis of TECs marker genes (*EOMES, GATA3, ELF5* in upper panel) and trophoblast marker genes (*CGA, CGB, GCM1, HLA-G* in lower panel) in hESC- day 4 TECs and day 9 trophoblasts. N=3. **g,** ELISA analysis of hCG in hESC-derived TECs and trophoblasts. N=3. Data are presented as mean+/-standard deviation. *P* values were calculated by unpaired two-tailed Student's t test. **P*< 0.05, ***P*< 0.01, and ****P*< 0.001. Source data for Supplementary Figure 1c and 1f-g are provided as a Source Data file.

Supplementary Figure 2

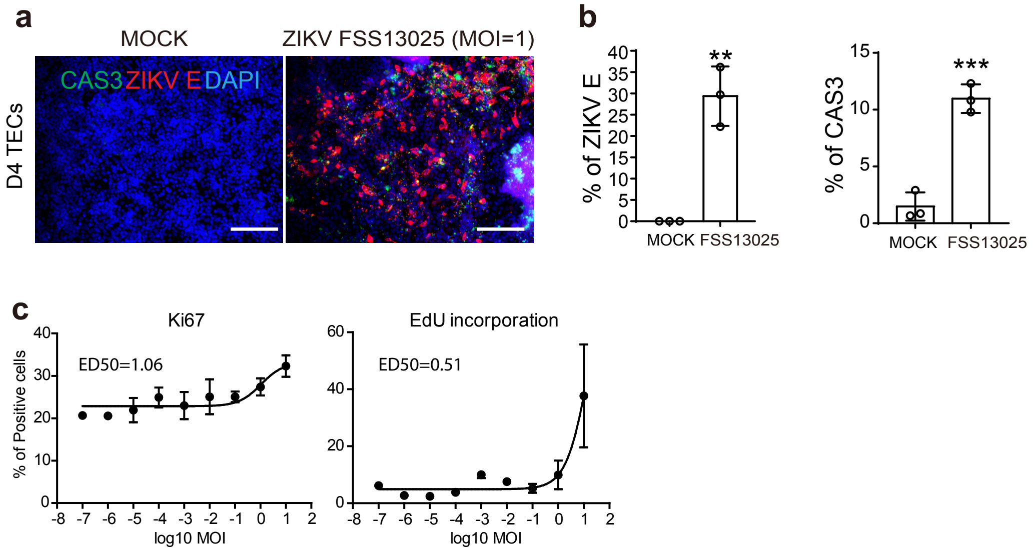

Supplementary Figure 2: Effects of ZIKV infection on TECs. Related with Figure 2.

a, Immunostaining of ZIKV E in TECs at 3 days post-infection with FSS13025 strain (MOI=1). Scale bar=100 µm. **b**, The percentage of ZIKV infected cells (left panel) and CAS3+ cells (right panel) were quantified in **a**. N=3. **c**, Quantification of the percentage of Ki67+ cells or EdU+ cells of TECs at 72 hpi with ZIKV (MR 766, MOI ranging from 10^-7 to 10^1). Data are presented as mean+/-standard deviation. *P* values were calculated by unpaired two-tailed Student's t test. $^{**}P < 0.01$, and $^{***}P < 0.001$. Source data for Supplementary Figure 2b are provided as a Source Data file.

Supplementary Figure 3

a

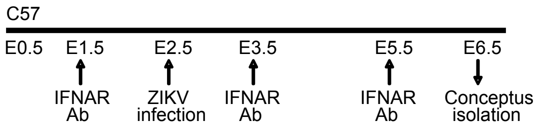

b

	MOCK	IFNAR Ab + MOCK	IFNAR Ab + ZIKV
# of Pregnant	8	5	8
# of Not pregnant	1	1	6
Total plugs	9	6	14
Pregnant/plugged rate	88.9%	83.3%	57.1%

c

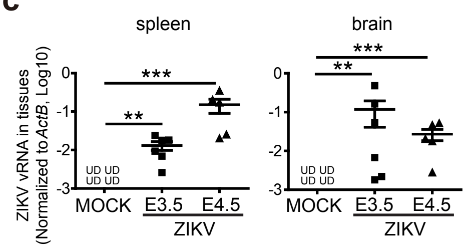

Supplementary Figure 3: ZIKV infection of mouse models. Related to Figure 4.

a, Scheme of ZIKV infection in pregnant C57 female mice treated with anti-IFNAR neutralizing antibody. **b,** Pregnancy rate of infected mice in **a**. **c,** qRT-PCR quantification of ZIKV vRNA in spleen and brain associated with Fig. 4a. N=4 for MOCK, N=6 for E3.5, and N=5 for E4.5. Data are presented as mean+/-standard deviation. *P* values were calculated by one-way repeated-measures ANOVA or two-way repeated-measures ANOVA with a Bonferroni test. $**P< 0.01$, and$***P< 0.001$. Source data for Supplementary Figure 3b-c are provided as a Source Data file.

Supplementary Table 1. Summary of the published studies on ZIKV and human placentas or trophoblasts.

Cells	Virus Strain	Virus Titre	PMID
Trophoblast progenitors (7.3 and 15.6 weeks)<br>Cytotrophoblasts (15.6-21.1 weeks) and (37.2 weeks)<br>Placental villus explants (7.5-8.4 weeks)	MR766	MOI=0.001-3	27443522
Placental microphage	PRVABC59	MOI=1	27247001
Human placenta villi, decidua, and fetal membrane explants from three donors (age 21–23 weeks gestation)	Paraiba 2015	5x10^6 FFU	29386359
Primary villous CTB<br>Trophoblasts derived from hESCs	MR766, FSS13025	MOI = 0.000027 to 1.08	28193876
Decidual and chorionic villus organ cultures	MP1751	5x10^4 per well in 48-well plates	27974560
Primary HC and trophoblasts dissected from membrane-free villous placenta	PRVABC59	MOI=1	28776046
human placental macrophages and placental villous fibroblasts	FSS13025, MexI-44 and MR766	MOI=1	27595140
Human placental (7-12 weeks)	ZIKV Brazil	6x10^10 RNA copies ml^-1	27759009
Human placental trophoblasts isolated from termed birth (37-41 weeks)	ZIKV-FLR	MOI=0.1-1	28128342
Human placental explants from second trimester (13-24 weeks gestation)	ZIKV Brazil	5x10^5 FFU ml^-1	28910635
Midgestation chorionic villi(17-23 weeks)	ZIKV-FLR	5x10^5 FFU ml^-1	28784796
Trophoblast cell lines<br>BeWo, JEG3, JAR, HTR8<br>Term placentas	MR766<br>FSS13025		27066743

JEG-3, BeWo, and HTR-8/SVneo cells	H/PF/2013	MOI=0.1	27180225
PHTs and hESC-derived trophoblasts	Paraiba 2015	MOI=1	29277269
HTR-8/SVneo	FSS13025	MOI=1 or 5	29331320
JAR and Vero cells,<br>Trophoblasts derived from hESCs	FSS13025, ib-H30656, MexI-44, MR766, PANcdc259249, SEN/1984/41525-DAK	MOI=0.01	29985932
Human placenta cell lines	PRVABC59	MOI=2	28685770
Jeg-3 cells	Mex2-81	MOI=0.5	28099856
Immortalized human trophoblast Sw.71 cells	FSS13025	MOI=20	27613665
Trophoblast cell line, BeWo	ZIKV Brazil	MOI=1	30003503
hESC-derived day 8 cytotrophoblast and syncytiotrophoblast<br>Primary cytotrophoblast from term birth<br>Syncytiotrophoblast differentiated primary cytotrophoblast	MR766	MOI=0.14-1.08	28193876
JEG-3	Paraiba 2015	MOI=0.1	28694387

Supplementary Table 2. Summary of the published *in vivo* studies of ZIKV infection during pregnancy.

Species	Stage	Virus titers	Route of Inoculation	PMID
Ifnar1$^{-/-}$ mice; WT mice+anti-ifnar	E6.5, E7.5	H/PF/2013 10^3 FFU	subcutaneous	27180225
Axl$^{-/-}$, *Mer*$^{-/-}$, *Axl*$^{-/-}$ *Mer*$^{-/-}$, *Axl*$^{-/-}$*Tyro3*$^{-/-}$ mice+anti-ifnar	E6.5	FSS13025 1.5X10^5 PFU<br>Brazil 10^4 FFU<br>MEX2-81 10^5 PFU	Vaginal<br>Intraperitoneal<br>Intracranially	28423319
Ifnar1$^{-/-}$ mice	E6, E9, E12	ZIKV Brazil<br>10^3 FFU	subcutaneous	28910635
Ifnar1$^{-/-}$ mice	E5.5, E10.5, E15.5	1X10^5 PFU	intraperitoneal	29117563
WT mice	E9.5	PRVABC59, 3.4X10^5PFU	intravenous	29634758
C57BL/6J mice	E8.5	PRVABC59, 1x10^4 PFU	intraperitoneal	30210488
C57BL/6 or SJL	E10–13	ZIKV Brazil<br>5x10^2, 8x10^9 or 2x10^11 PFU ml^-1	intravenous	27279226
C57BL/6NCrl, *Rag2*$^{-/-}$, *Ifnar1*$^{-/-}$,*Irf3*$^{-/-}$*Irf7*$^{-/-}$, *Tlr7*$^{-/-}$*Mx1*, *Mavs*$^{-/-}$*Mx1* and *Tlr7*$^{-/-}$*Mavs/ Mx1*, *Mb21d*$^{-/-}$	E4.5 or E8.5	FSS13025, 2.5x10^4 to 5.2x10^5 PFU,<br>Or FSS13025, 1.5x10^5 PFU	Intravaginal infection<br>intraperitoneal	27565347
C57BL/6J or 129S1/SvImJ	E14.5	MEX1-44, 1.7x10^3 TCID50	to embryonic brain	27729407
Ifnar1$^{-/-}$ female X C57BL/6 male<br>Or<br>C57BL/6 female X C57BL/6 male, treated with 1 mg	E5.5	ZIKV-Dakar, 1x10^3 FFU	subcutaneous	27819683

injection of anti-Ifnar1 (MAR1-5A3)				
FVB/NJ and C57BL/6J	5.5, 7.5, or 9.5 dpc	HS-2015-BA-01, 1x10^3 to 1x10^8 PFU	intravenous	28231241
Ifnar1$^{-/-}$	Breed with infected male	MEX2-81, 1x10^5 PFU		28472297
C57BL/6	E15	SZ01, 250 and 500 PFU	Intra-amniotic Injection	28583742
Rag1$^{-/-}$	7dpm	Paraiba 2015, 1x10^4 PFU	intraperitoneal	28775298
WT or Atg16l1flox/flox X Cyp19-Cre	E9.5	Paraiba 2015, 1x10^3 FFU	subcutaneous	28694387
Ifnar1$^{-/-}$or<br>WT treated with anti-Ifnar1 blocking mouse mAb (MAR1-5A3)	E6, E9, or E12	Paraiba 2015, 1x10^3 FFU	subcutaneous	28910635
Ifnar1$^{-/-}$females X WT males<br>Or<br>WT treated with anti-Ifnar1 blocking mouse mAb (MAR1-5A3)	E6.5	Paraiba 2015, 1x10^3 FFU	subcutaneous	28945244
Ifnar1$^{-/-}$	E5.5, E10.5 and E15.5	FSS13025, 1x10^5 PFU	intraperitoneal	29117563
Ifnar1$^{-/-}$female X *Ifnar1*$^{+/-}$male	E5.5 and E 8.5	FSS13025, 1.5x10^5 PFU	intravaginal	29305462
C57BL/6J	E9.5 or E12.5	PRVABC59, 3.4x10^4, 1x10^5, or 3.4x10^5 PFU	intravenous	29634758
C57BL/6J or 129 mice	E14.5	MEX-1-44 or MR766, 3.4×10^2 TCID50	Intracranially	28993398

hSTAT2 KI or WT female X hSTAT2 KI or WT female	E6.5	Dakar 41525, 1x10^4 to 1x10^6 PFU	subcutaneous	29746837
Rhesus macaques	31 days of gestation (dGA)<br>51 dGA<br>115 dGA	PRVABC59, 1X10^5 FFU	subcutaneous	29343712
Macaque monkeys	GD79, GD83 (~human 14 weeks)<br>GD68, GD72 (~human 9 weeks)	PRVABC59, 1x10^5 FFU<br>FSS13025, 1x10^7 PFU<br>SPH20152, 5x10^5 PFU	subcutaneous<br>subcutaneous<br>subcutaneous	29967348

Supplementary Table 3. Summary of the ZIKV titer in semen, testes and blood of human, mouse and non-human primates.

Species	Tissue type	Viral titer or vRNA copy number	Method of measurement	PMID
Human	Semen	2.9x10^7 ml^-1 and 1.1x10^7 ml^-1	QPCR	25625872
Human	Semen	2.9x10^8 ml^-1 and 3.5x10^7 ml^-1	QPCR	27074370
Human	Semen	Up to 5x10^4 ml^-1, and then ranging from 2x10^3 to 1x10^4 ml^-1	QPCR	27542178
Human	Semen	Up to ~6.5x10^8 ml^-1	QPCR	30405055
Human	Semen	1.4x10^7 ml^-1	QPCR	30059980
Human	Serum	2.8x10^5 ml^-1	QPCR	30059980
Human	Semen	Ranging from 1x10^7 to 2.5x10^8 ml^-1	QPCR	29641964
Human	Semen	Ranging from 8.03x10^3 to 2.55x10^6 ml^-1	QPCR	28711704
Human	Serum	Up to ~2.4x10^4 ml^-1	QPCR	28838639
Human	Semen	Up to ~1.5x10^10 ml^-1	QPCR	28838639
Human	Semen	Up to ~1.6x10^9 ml^-1	QPCR	28813216
Human	plasma	Up to ~4.5x10^2 ml^-1	QPCR	29300893
Human	Whole blood	Up to ~5.0x10^4 ml^-1	QPCR	28838639
Human	Blood	Up to ~7.9x10^3 ml^-1	QPCR	28813216
Rhesus and cynomolgus macaques	Semen	ranging from 3.4x10^4 to 1.1x10^6 ml^-1	QPCR	27694931
Rhesus macaques	Plasma	Ranging from 1.1x10^4 to 7.4x10^4 ml^-1	QPCR	28667164
Rhesus macaques	Semen	Up to ~2.2x10^3 ml^-1	QPCR	28667164
AG129 mice	Semen	Up to ~6.3x10^6 gram^-1 tissue	plaque assay	28199846
AG129 mice	Semen	Ranging from 1.3x10^4 to 2.5x10^6 ml^-1	QPCR	28985234

AG129 mice	seminal vesicle	Up to ~5.0x10^2 gram^-1 tissue	plaque assay	28199846
AG129 mice	Testes	Up to ~2.5x10^4 gram^-1 tissue	plaque assay	28199846
AG129 mice	Testes	Ranging from 1.0x10^8 to 2.0x10^8 gram^-1 tissue	plaque assay	30070988
AG6 mice	Semen	~1x10^8 gram^-1 total RNA	QPCR	28680856
Ifnar1$^{-/-}$mice	Semen	Ranging from 1x10^6 to 1x10^10 gram^-1 of total RNA	QPCR	28589934
Ifnar1$^{-/-}$mice	Semen	Up to ~1.1x10^5 ml^-1	QPCR	30053014
Ifnar1$^{-/-}$mice	Testes	Up to ~1x10^5 gram^-1 tissue	plaque assay	28261663
Ifnar1$^{-/-}$mice	Testes	Ranging from 1.5x10^3 to 6.6x10^5 gram^-1 tissue	QPCR	30053014
Ifnar1$^{-/-}$mice	Testes	Ranging from 3x10^7 to 1x10^12 gram^-1 of total RNA	QPCR	28589934

Supplementary Table 4. Summary of sample ID used in clustering analysis acquired from public data source.

Figure of presence	accession number of data set	sample group	sample name in Figure	accession number of sample
Fig. 2i	GSE98423	*Ifnar*$^{+/-}$, uninfected	1_U20_1	GSM2595175
			2_U20_2	GSM2595179
			6_U20_9	GSM2595180
		Ifnar$^{-/-}$, uninfected	3_U20_3	GSM2595176
			4_U20_4	GSM2595181
			5_U20_8	GSM2595182
		Ifnar$^{+/-}$, infected	19_ZV108_1	GSM2595177
			23_ZV108_5	GSM2595185
			25_ZV108_8	GSM2595186
			17_ZV105_4	GSM2595187
			18_ZV105_5	GSM2595188
		Ifnar$^{-/-}$, infected	20_ZV108_2	GSM2595178
			14_ZV105_1	GSM2595183
			15_ZV105_2	GSM2595184
			22_ZV108_4	GSM2595189
			24_ZV108_7	GSM2595190
Supplementary Fig. 1d	GSE66507	EPI2	EPI2_1	GSM1868817
			EPI3_1	GSM1624228
			EPI4_1	GSM1868818
			EPI4_2	GSM1868820
			EPI4_3	GSM1868823
			EPI5_1	GSM1868819
			EPI5_2	GSM1868822
			EPI6_1	GSM1868821
		TE2	TE3_1	GSM1624224

			TE3_2	GSM1624226
			TE3_3	GSM1624225
			TE3_4	GSM1624227
			TE3_5	GSM1624223
			TE3_6	GSM1868815
			TE4_1	GSM1868816
			TE8_1	GSM1624230
			TE8_2	GSM1624236
			TE8_3	GSM1624235
			TE8_4	GSM1624222
			TE8_5	GSM1624233
			TE8_6	GSM1624231
			TE8_7	GSM1624229
			TE8_8	GSM1624234
			TE9_1	GSM1624237
	GSE36552	EPI1	R1	GSM922216
			H8	GSM922213
			H1	GSM922206
			H2	GSM922207
			H3	GSM922208
		TE1	G13	GSM922194
			G2	GSM922195
			G3	GSM922196
			G4	GSM922197
			G5	GSM922198
			G6	GSM922199
			G7	GSM922200
			G8	GSM922201
			G9	GSM922202

			G10	GSM922203
			G11	GSM922204
			G12	GSM922205
			H4	GSM922209
			H5	GSM922210
			H6	GSM922211
			H7	GSM922212
			H9	GSM922214
			H10	GSM922215

Supplemental Table 5. Primers used for quantification of human and mouse genes.

Gene names	Forward primer	Reverse primer
GATA3	ACATGCTGACCACGCCCACG	GCAGGGCTCTAACCCATGGC
FOXD3	GGGCAAGGGCAACTACTGGA	GTAAGCGCCGAAGCTCTGCAT
EOMES	AGCTCTCCAAGGAGAAAGTG	GCCTTCGCTTACAAGCACTG
CDX2	CCCTCGGCAGCCAAGTGAAA	TCCTCCGGATGGTGATGTAG
TEAD4	AGACCTTGCTGTGCATTGCC	CTCCCCGAGTCTCTCATTCT
ELF5	TAGGGAACAAGGAATTTTTCG GG	GTACACTAACCTTCGGTCAACC
GCM1	AAGAAGTCCTGCCTGGGTGT	CGTGCCTCCAGAAGTTGGT
CGA	CAACCGCCCTGAACACATCC	CAGCAAGTGGACTCTGAGGTG
CGB	TGAGATCACTTCACCGTGGTC TCC	TTTATACCTCGGGGTTGTGGGG
HLAG	CAGATACCTGGAGAACGGGA	CAGTATGATCTCCGCAGGGT
Human Beta-ACTIN	ACCTTCTACAATGAGCTGCG	CCTGGATAGCAACGTACATGG
Bax	CTGACATGTTTGCTGATGGC	GAAGTCCAGTGTCCAGCC
Bak1	GGAGCAGAGTCATTCAGGTG	CTCTGTGTCCTGGGCAAC
Bad	AGGATGAGCGATGAGTTTGA G	CCTTTGCCCAAGTTTCGATC
Bim	GTTCAACGAAACTTACACAAG GAG	GTATCCTGGCTGCATGTAGATC
Puma	ACCTCAACGCGCAGTAC	CCTAGTTGGGCTCCATTTCTG
Mouse Beta-Actin	ACCTTCTACAATGAGCTGCG	CTGGATGGCTACGTACATGC

Supplemental Table 6. Primer/probe sets used for quantification of ZIKV genome.

Gene names	Forward primer	Reverse primer
ZIKV vRNA-RT	*TACTTGTACAGCTCGTCCATG*CCACTAACGTTCTTTTGCAGACAT	
ZIKV vRNA-PCR	CCGCTGCCCAACACAAG	TACTTGTACAGCTCGTCCATG
ZIKV vRNA-PCR probe	5'-/56-FAM/AGCCTACCT/ZEN/TGACAAGCAATCAGACACTCAA/3IABkFQ/-3'	
Human ACTB-RT	CCTGGATAGCAACGTACATGG	
Human ACTB-PCR	CCTTGCACATGCCGGAG	ACAGAGCCTCGCCTTTG
Human ACTB-PCR probe	5'-/5HEX/TCATCCATG/ZEN/GTGAGCTGGCGG/3IABkFQ/-3'	
Mouse Actb –PCR	ATGCCGGAGCCGTTGTC	GCGAGCACAGCTTCTTTG
Mouse Actb –PCR probe	5'-/5HEX/CCGCCACCA/ZEN/GTTCGCCATG/3IABkFQ/-3'	
