# Supplementary material for: Pre- and peri-implantation Zika virus infection impairs fetal development by targeting trophectoderm cells
Source: Nat Commun. 2019 Sep 13;10:4155. doi: 10.1038/s41467-019-12063-2 (PMC6744420; doi:10.1038/s41467-019-12063-2)
Supplement: Supplementary file 2 — Description of Additional Supplementary Files [file 41467_2019_12063_MOESM2_ESM.pdf]

**Description of Additional Supplementary Files**

File Name: Supplementary Movie 1
Description: ZIKV infection (ZIKV E, red) and cell apoptosis (Cleaved Caspase 3/CAS3, white) in mouse trophectoderm (CDX2, green) was indicated by immunostaining at 24 hours post infection in MOCK infected mouse blastocysts.

File Name: Supplementary Movie 2
Description: ZIKV infection (ZIKV E, red) and cell apoptosis (Cleaved Caspase 3/CAS3, white) in mouse trophectoderm (CDX2, green) was indicated by immunostaining at 24 hours post infection with MR766 strain ZIKV (2X10^4 IFU ml^-1) infected in mouse blastocysts.

File Name: Supplementary Movie 3
Description: ZIKV infection (ZIKV E, red) and cell apoptosis (Cleaved Caspase 3/CAS3, white) in mouse trophectoderm (CDX2, green) was indicated by immunostaining at 24 hours post infection with MR766 strain ZIKV (6X10^4 IFU ml^-1) in mouse blastocysts.
